# Processing and Characterization of Spark Plasma Sintered SiC-TiB_2_-TiC Powders

**DOI:** 10.3390/ma15051946

**Published:** 2022-03-05

**Authors:** Sergey N. Grigoriev, Yuri Pristinskiy, Thet Naing Soe, Alexander Malakhinsky, Mikhail Mosyanov, Pavel Podrabinnik, Anton Smirnov, Nestor Washington Solís Pinargote

**Affiliations:** 1 Laboratory of Electric Current Assisted Sintering Technologies, Moscow State University of Technology “STANKIN”, Vadkovsky per. 1, 127055 Moscow, Russia; s.grigoriev@stankin.ru (S.N.G.); y.pristinskiy@stankin.ru (Y.P.); m.mosyanov@stankin.ru (M.M.); p.podrabinnik@stankin.ru (P.P.); 2 Department of High-Efficiency Machining Technologies, Moscow State University of Technology “STANKIN”, Vadkovsky per. 1, 127055 Moscow, Russia; kothetnaingsoe6151@gmail.com (T.N.S.); a.malakhinsky@gmail.com (A.M.)

**Keywords:** SiC-TiB_2_-TiC composites, cutting tool material, spark plasma sintering, mechanical properties, microstructure

## Abstract

SiC-TiB_2_-TiC composites with matrices consisting of semiconductor material (SiC), conductive materials (TiB_2_-TiC), or their combination were fabricated by spark plasma sintering (SPS) at 2000 °C in a vacuum under a pressure of 80 MPa for 3 min. The composition and microstructure of the obtained composites were studied by X-ray diffraction and a scanning electron microscope equipped with an energy-dispersive detector. The flexural strength, Vickers hardness, and fracture toughness of SPSed samples were determined. Based on the observations in this work, three variations of the sintering process were proposed with different matrix compositions. The dense (99.7%) 60SiC-25TiB_2_-15TiC vol.% sintered ceramic composites exhibited the highest strength and hardness values of the studied composites, as well as a fracture toughness of 6.2 MPa·m^1/2^.

## 1. Introduction

Currently, advanced ceramics are widely used in cutting tools, drawing or extrusion, seal rings, valve seats, bearing parts, a variety of high-temperature engine parts, etc. [1,2,3,4]. In most of these applications, silicon carbide (SiC), titanium carbide (TiC), and titanium diboride (TiB_2_) are extensively applied, because they are considered promising materials for high-temperature components with structural and wear resistance as a result of their intrinsic characteristics, as well as their exceptional combination of electrical and mechanical properties [5].

Silicon carbide is a well-known ceramic material with outstanding properties, such as low density and high hardness [6], excellent corrosion and oxidation resistance, high strength (even at high temperatures) [7,8,9], high thermal conductivity, a low thermal expansion coefficient [10,11,12], and good thermal shock resistance [13].

However, despite the properties mentioned above, monolithic SiC materials show relatively low fracture toughness, which, combined with the well-known machining difficulties (chipping) arising from their brittleness and high hardness, is a barrier to their wide implementation in advanced engineering applications. In order to improve the mechanical properties and increase the wear resistance of SiC-based composites, different reinforcing phases, such as TiC and TiB_2_, have been used [14,15].

Titanium carbide has attracted considerable attention because it is a lightweight, inexpensive, ultra-high-temperature ceramic material with high hardness and wear resistance [16,17], good thermal and electrical conductivity, a high melting point, and extreme thermal and chemical stability [18], and it also shows a higher elastic modulus and thermal expansion coefficient than SiC [19,20]. The combination of these characteristics makes this material a promising strengthening component for SiC-based composites. For instance, the introduction of TiC completely hinders the growth of elongated SiC grains and ensures the formation of a more equiaxed microstructure [21], which leads to an increase in the strength of the material [22]. On the other hand, some studies have shown that the introduction of TiC particles is an effective way of increasing the fracture toughness of the SiC matrix [23]. This can be explained by the large difference in thermal expansion coefficients between reinforcing phases and the SiC matrix, which causes significant radial tensile stresses at grain boundaries and circular tangential compressive stresses in the matrix [24]. The presence of these stresses makes crack deflection, crack branching, and microcracking possible, resulting in increased fracture toughness.

Titanium diboride is commonly known for its excellent physical, mechanical, and chemical properties, such as its exceptional hardness, wear and corrosion resistance [25], and high thermal and electrical conductivities [26], making TiB_2_ an attractive reinforcement material for cutting tools and wear-resistant parts based on SiC ceramic [27]. Furthermore, TiB_2_ is also used as a phase in cutting tools, in which it serves the functions of lubrication and crack healing due to its transformation to TiO_2_ and B_2_O_3_ under the action of the cutting heat [28]. Thus, the inclusion of TiB_2_ in SiC-TiC composites can give the material both a toughening effect and a self-healing function. Moreover, the addition of TiB_2_ to SiC-TiC composites is expected to increase the wear resistance of the ceramic cutting tool and thus expand its implementation to advanced engineering applications [28]. Some characteristics of SiC, TiC, and TiB_2_ are shown in Table 1.

**Figure 1:**
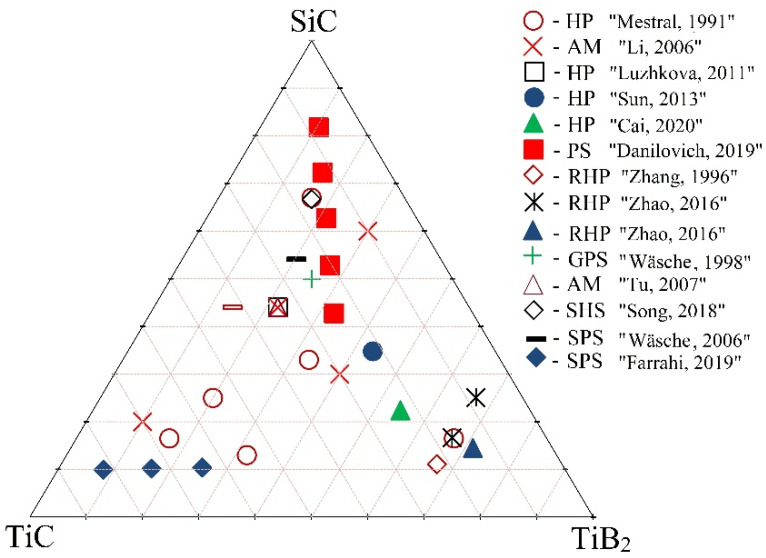
Phase diagram of the SiC-TiB_2_-TiC system (mol%) indicating the studied compositions in previous works [5,9,44,45,46,47,48,49,50,51,52,53,54,55].

For all of the above reasons, the SiC-TiB_2_-TiC system can be an excellent composite material for cutting tool applications, which combines all of the necessary component properties, such as lightweight and high mechanical, thermal, electrical, and tribological properties [41,42,43], which have been confirmed by previous studies in this system (Figure 1).

Figure 1 shows the SiC-TiB_2_-TiC phase diagram (mol%) and indicates the studied compositions in previous works. In addition, the used sintering methods and their references are shown. From this picture, it is clear that hot pressing (HP) has been the most popular consolidation method for this system. For instance, De Mestral and Thevenot [5] sintered the TiB_2_-TiC-SiC system by hot pressing at 2200 °C with different amounts of phases and showed that it is possible to reach a relative density of 99.8% and to increase both fracture toughness and strength to 6.7 MPa·m^l/2^ and 1070 MPa, respectively. In addition, the authors determined that the critical flaw size ranges from 6.2 µm to 17.7 µm.

Zhang et al. [48] used reactive hot pressing at 2000 °C in order to sinter the 11.11TiC–66.71TiB_2_–22.18SiC (mol.%) composite using TiH_2_, Si, and B_4_C as starting materials. In this study, the best relative density achieved was 99.8%. Moreover, the maximum values of fracture strength and fracture toughness were 680 MPa and 6.9 MPa·m^l/2^, respectively.

Li et al. [9] synthesized TiC-TiB_2_-SiC composites by arc melting using TiC, TiB_2_, and SiC powders as starting materials in an Ar atmosphere, in which the samples were melted and solidified two times. In this work, the authors determined that the eutectic composition was 44SiC-22TiB_2_-34TiC (mol%). The TiC-TiB_2_-SiC eutectic composite showed a hardness of 25.5 GPa and electrical and thermal conductivities of about 2.81 × 10^−3^ S/m and 60 W/K·m, respectively.

In his Ph.D. thesis, Danilovich [47] obtained different compositions of the SiC-TiB_2_-TiC system by pressureless sintering (PS) at 2100 °C, 2150 °C, and 2220 °C in an argon atmosphere. The author established that the composite 60SiC-25TiB_2_–15TiC (vol.%) sintered at 2015 °C showed the best mechanical properties (relative density of 98.9%, bending strength of 387 MPa, Vickers hardness of 23.04 GPa, and fracture toughness of 8.87 MPa·m^l/2^).

The consolidation of the SiC-TiB_2_-TiC system by traditional sintering routes requires higher temperatures or higher pressures for long holding times due to the covalent bonds, high melting temperatures, and low self-diffusion coefficients of the system components. A promising method for the compaction of this system is spark plasma sintering (SPS).

In spark plasma sintering, heating is achieved by passing a pulsed DC electrical current through the matrix when the sample is nonconductive or through the sample when it is conductive. This gives the SPS method certain advantages, such as high heating rates (from 100 °C/min to 1000 °C/min) that permit faster densification, which allows sintering at lower temperatures and also with very short cycles, reducing grain growth [56,57,58,59,60]. Moreover, the possibility of applying external pressure during the sintering process makes it possible to carry out the consolidation of poorly sinterable ceramics in very short times, producing materials with better mechanical properties [61,62].

In the literature, it is possible to find a few works related to the sintering of the SiC-TiB_2_-TiC system by SPS. For instance, Wäsche et al. [54] studied the oscillating sliding wear behavior of the 52SiC-24TiC-24TiB_2_ (mol.%) composite. However, the authors did not provide any information regarding the sintering regimes used to obtain the composite.

Recently, Fattahi et al. [55] studied the consolidation of the TiC-TiB_2_-SiC system by SPS. In this study, SiC whiskers (SiC_w_) were used instead of common SiC particles. The samples were compacted at 1900 °C with a dwell time of 7 min at 40 MPa. The best relative density (around 96%) and flexural strength (about 420 MPa) were obtained for the specimen with 10 vol% TiB_2_ content. On the other hand, the most significant Vickers hardness (27.67 GPa) was measured in the sample with 30 vol% TiB_2_.

The aim of the present work was to use spark plasma sintering to obtain a SiC-TiB_2_-TiC system with matrices consisting of semiconductor material (SiC), conductive materials (TiB_2_-TiC), or their combination in order to understand the influence of the current flow through the sintering zone and heat generation during the consolidation of each material, as well as to provide a representation of the sintering process for each case. The results of this study were compared with composites obtained by pressureless sintering (PS) at different temperatures, which were studied in [47]. The choice of this study [47] was made for two reasons: the composites contain semiconductor and conductive matrices, as well as a combination of both, and the mechanical properties of the composites obtained by PS are provided.

## 2. Materials and Methods

### 2.1. Processing

Commercially available SiC, TiC, and TiB_2_ powders that were used as raw materials in this study are presented in Table 2.

Thus, the composites were prepared by adding TiB_2_ and TiC in total amounts that varied from 20 vol.% (for the semiconductor matrix) to 60 vol.% (for the conductive matrix) (Table 3).

Raw powders were mechanically mixed in an ML1 ball mill (Zapadpribor, Kaluga, Russia) for 36 h in a 250 mL polyethylene jar with SiC balls (∅3 mm) at a rotation speed of 250 rpm. Isopropanol was used as dispersion medium, and the powder-to-ball weight ratio was 1:3. The obtained wet mixtures were dried at 70 °C for 12 h in air. The resulting powders were ground in an agate mortar and subsequently passed through a 63 μm sieve.

### 2.2. Spark Plasma Sintering

Powder mixtures were sintered in an H-HP D 25 SD spark plasma sintering machine (FCT Systeme GmbH, Rauenstein, Germany), and samples (diameters of 20 and 45 mm; ~5 mm in height) were fabricated. First, a dilatometric test was carried out on the SPS machine with the minimum possible pressure (43 MPa) to determine the temperature at which the highest density of the samples occurred. It was noted that the maximum compaction took place at approximately 2000 °C, regardless of the composition of the studied system. In this work, sintering was carried out at 2000 °C and 80 MPa in vacuum. A minimum pressure of 43 MPa was maintained until a temperature of 300 °C was obtained, and then pressure and temperature were continuously increased until 1900 °C with a heating rate of 100 °C/min. Then, a heating rate of 25 °C/min was applied to reach the maximum temperature, which was maintained for 3 min (dwell time). After the sintering process, the samples were cooled naturally in the sintering chamber.

### 2.3. Microstructural Characterization

Sintered SiC-TiB_2_-TiC samples were polished using a conventional polishing procedure with up to 1 μm diamond suspension. The microstructures, fracture surfaces, and cracks formed after indentation were observed by a VEGA 3 LMH scanning electron microscope (SEM) (Tescan, Brno, Czech Republic) equipped with X-Act EDX detector (Oxford Instruments, Abingdon, UK) for energy-dispersive spectroscopy (EDS) in order to analyze the element distribution of the material. Energy-dispersive analysis was carried out at HV = 20 kEV to induce all of the characteristic lines of the elements. The resolution of the detector (full width at half maximum, FWHM) is 127 eV. The collection and processing of spectra were carried out in the AZtec software (Oxford Instruments, Abingdon, UK). During the analysis, the average values of the concentration of elements were determined with confidence intervals of 3σ.

Phase identification of sintered samples and raw powders was carried out using an Empyrean diffractometer (PANalytical, Almelo, Netherlands) with a Cu–Kα radiation source (λ = 1.5405981 Å) in the 2θ angle range of 25–75°.

### 2.4. Density and Mechanical Properties

Bulk densities of sintered samples were determined using the Archimedes method in distilled water. Theoretical densities were calculated according to the rule of mixtures assuming densities of 3.26 g/cm^3^ for SiC, 4.85 g/cm^3^ for TiC, and 4.35 g/cm^3^ for TiB_2_, which were measured by an AccuPyc II 1340 helium pycnometer (Micrometrics, Norcross, GA, USA). The relative density of the composites was then calculated as the bulk density divided by theoretical density.

Flexural strength was determined following the standard ASTM C1161-13 [63] using a displacement rate of 0.5 mm/min in an ElectroPuls E10000 universal testing machine (Instron, High Wycombe, UK) by three-point bend tests using bars with a dimension of 3 mm × 4 mm × 40 mm, and a span of 30 mm. Bars were ground and polished before testing, and two edges on the tensile surface were chamfered to an angle of 45° in order to eliminate a failure initiated from the edge of the specimen. For each composition, 4 samples were tested, and the arithmetic mean of the test results was calculated.

Vickers hardness (*H_v_*) of the samples was measured by the indentation method on the polished surfaces using a universal microhardness tester (Qness, Salzburg, Austria) with a standard diamond pyramid indenter. The load was set to 98 N, and the loading time was 10 s. A minimum of 10 indentations were made, and then the arithmetic mean of the test results for each composition was calculated.

The equation used for the hardness calculation was:

(1) $$H_{V}=\frac{0.1891\;P}{d^{2}}$$

where *P* is the set load (N), and *d* is the average length of two diagonals (mm).

Fracture toughness of the samples was measured by the microindentation method on the polished surfaces with an applied load of 98 N for 10 s. After indentation, the samples were washed in an ultrasonic bath in isopropanol prior to SEM observation. Fracture toughness was calculated using the formula given by Miranzo and Moya [64].

When the average length of cracks “*c*” (mm) to the average length of the two diagonals “*d*” (mm) ratio (*c/d*) is greater than 2.8, then Formula (2) is used to calculate the fracture toughness value.

(2) KIC=0.047·P(d0.42·c1.08)·[f(EHv)]

On the other hand, when the ratio (*c/d*) is less than 2.8, then Formula (3) is used to calculate the fracture toughness value.

(3) KIC=0.0232·P(d·c12)·[f(EHv)]

The function *f*(*E*/*Hv*) in both Formulas (2) and (3) is calculated by Formula (4):

(4) [f(EHv)]=0.768·(EHv)0.05+0.612·Ln(EHv)−2

where *H_v_* is the Vickers hardness of material (GPa), *P* is the applied load (N), and E is the Young’s modulus of material (GPa).

## 3. Results and Discussion

### 3.1. Relative Densities of Sintered Samples

**Figure 2:**
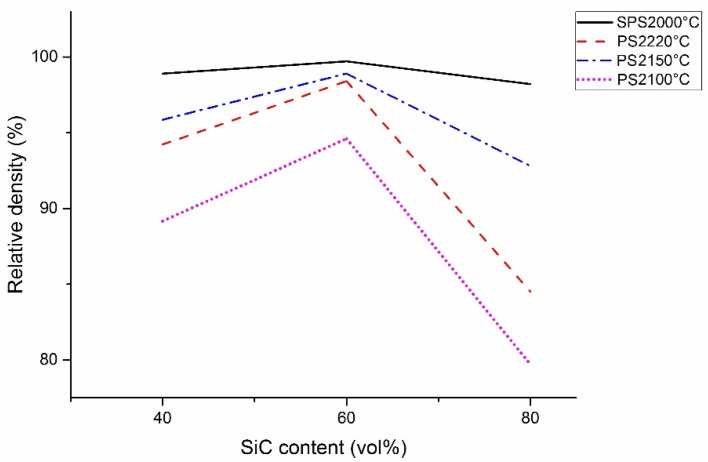
Relative density comparison of sintered SiC-TiB_2_-TiC composites by SPS and PS [47].

Figure 2 shows the relative densities of the SiC-TiB_2_-TiC composites obtained in this work and the values from [47]. As can be seen in this graph, the relative density tendency is very similar for both sintering methods.

For each temperature, the minimum density is observed in the 80SiC samples, while the 60SiC samples reach the maximum value. It can be seen that the highest relative density (99.7%), as well as the smallest difference between the maximum and minimum values (only 1.5%), occurs with SPS. PS samples have a lower relative density and larger differences (14.9%, 6.1%, and 13.9% at 2100 °C, 2150 °C, and 2220 °C, respectively) in comparison with SPS composites.

### 3.2. Microstructural Characterization

**Figure 3:**
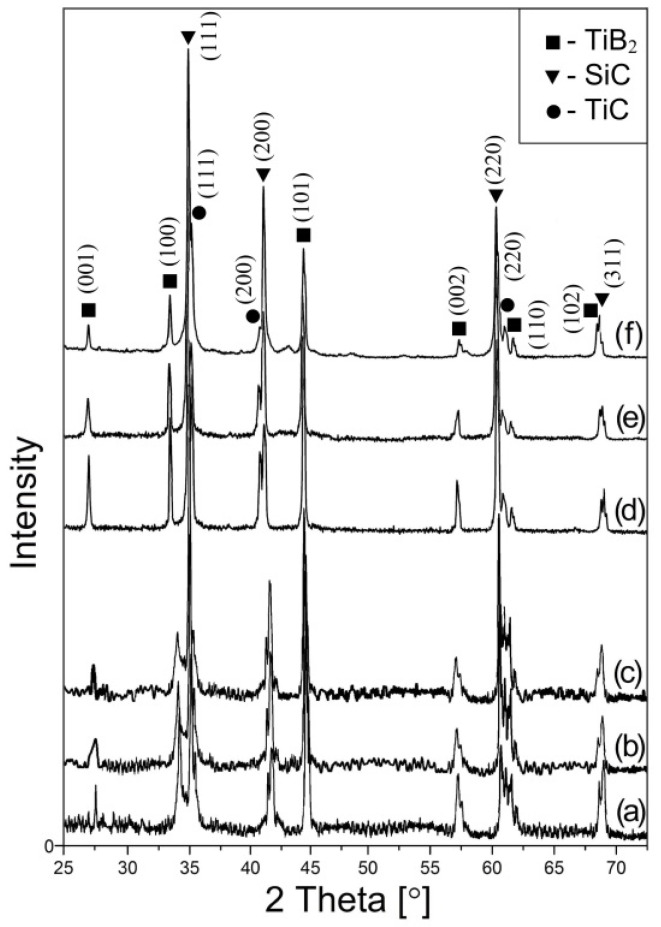
XRD pattern of as-prepared and sintered (at 2000 °C) SiC-TiB_2_-TiC composites with 40 vol.% (a,d), 60 vol.% (b,e) and 80 vol.% (c,f) SiC, respectively.

Figure 3 shows the XRD pattern of as-prepared and sintered SiC-TiB_2_-TiC composites in this work with 40 vol.% (a, d), 60 vol.% (b, e) and 80 vol.% (c, f) SiC, respectively. The patterns reveal that neither contamination nor the presence of secondary reactions occurred during the sintering of the powder. The database used for phase identification were PDF#29-1129, PDF#35-0741, and PDF#32-1383 for SiC, TiB_2_, and TiC, respectively.

**Figure 4:**
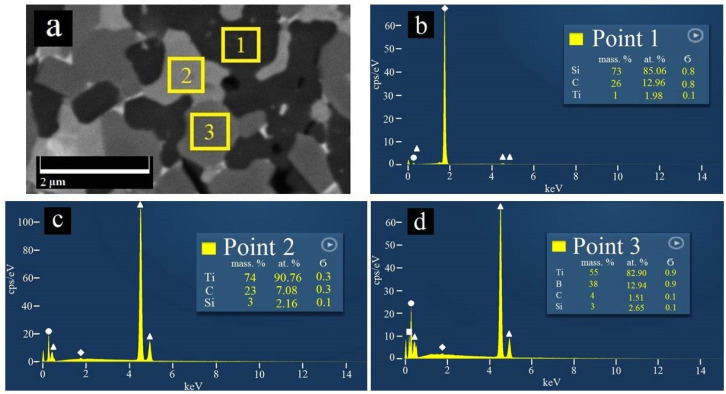
EDS analysis of 60 SiC composite: (**a**) SEM micrograph of polished surface, in which points 1, 2 and 3 show the places, where the spectra indicated in (**b**–**d**) were obtained, (**b**) the SiC spectrum, (**c**) the TiC phase, and (**d**) the TiB_2_ material. In the images in (**b**–**d**), the elements Si, Ti, C, and B are represented by white rhombuses, triangles, circles, and squares, respectively.

During SEM analyses of the sintered samples, three phases with different colors were observed. EDS results (Figure 4) confirm that the dark gray phase is SiC, the gray phase represents TiB_2_, and the light gray phase is TiC, with is in concordance with [9].

**Figure 5:**
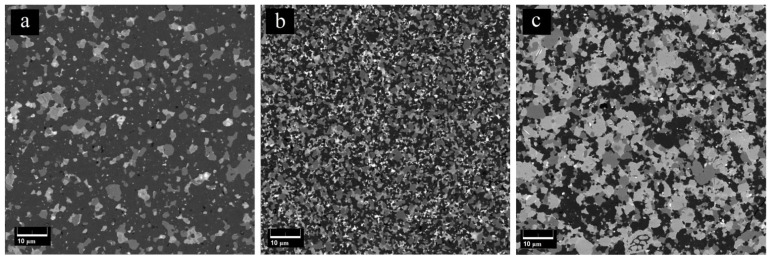
SEM images of the polished surfaces of (**a**) 80SiC, (**b**) 60SiC, and (**c**) 40SiC composites obtained by SPS.

**Figure 6:**
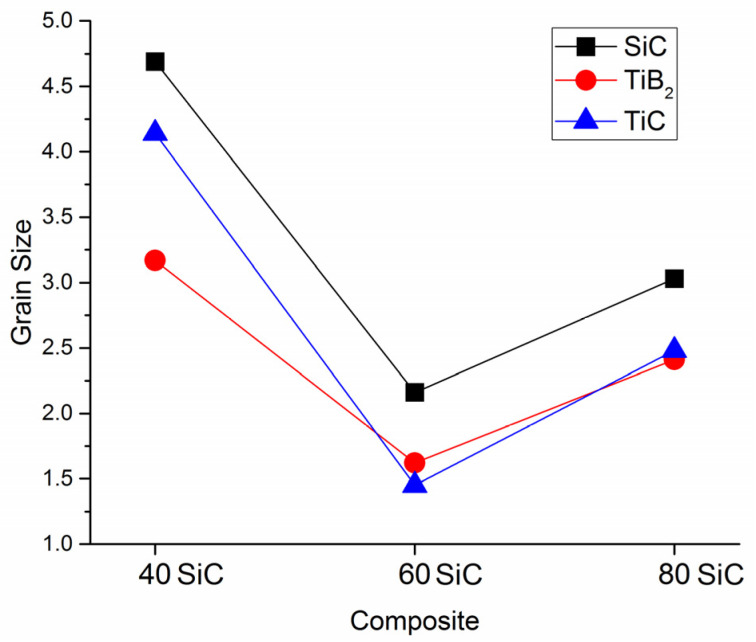
Average grain size (μm) of SiC, TiB_2_, and TiC in the 80SiC, 60SiC, and 40SiC composites.

In Figure 5, the SEM images of the polished surfaces of sintered composites are shown. Each composite has a different microstructure with a uniform distribution of the different phases. Figure 5a reveals the SEM micrograph of the composite with 80 vol.% SiC. It is clearly observed that, in this case, SiC forms the matrix of the composite, and the other two phases form globular grains within it. The phases’ grain sizes range from 2.41 to 3.03 μm (Figure 6), whereas the grain sizes of 80SiC obtained by PS are greater than 5 microns [47].

Figure 5b shows the surface of the 60SiC composite. This sample has the most homogeneous microstructure and the smallest grain size compared to the other two studied compositions. In this structure, the phases are homogeneously distributed and have the smallest grain size (Figure 6). In addition, the presence of pores is not observed in this structure.

Figure 5c demonstrates the microstructure of the 40SiC composite. From this figure, it is clearly observed that TiC and TiB_2_ form the composite matrix, and SiC forms agglomerates. In this figure, the presence of some pores is noted, but to a lesser extent than in the 80SiC sample. It is observed that TiC wets the edge of SiC grains very well.

**Figure 7:**
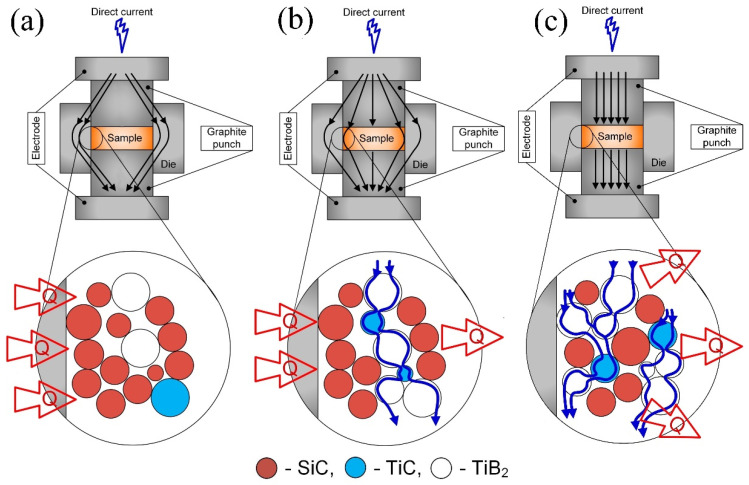
Schemes of the processes that take place during SPS of (**a**) 80SiC, (**b**) 60SiC, and (**c**) 40SiC composites.

We assume that during the consolidation of the 80SiC composite, in which the matrix is a semiconductor material (SiC), direct current pulses bypass the composite material and flow through the graphite punches and matrix (Figure 7a), in which heat is generated due to Joule heating. In this scheme, the sintering proceeds as in a conventional hot pressing, in which sintering will be delayed, since more time and energy are needed to heat mainly the graphite parts and then the powder.

**Figure 8:**
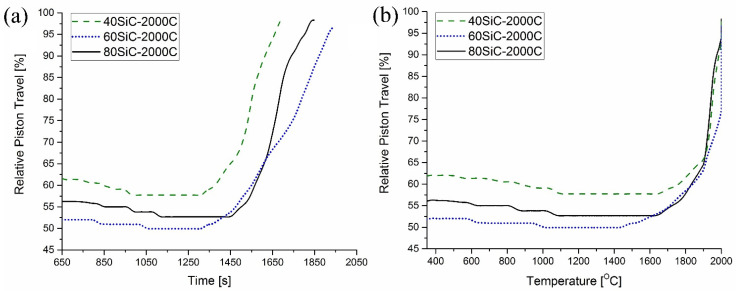
Relative piston travel vs. (**a**) time and (**b**) temperature of 80SiC, 60SiC, and 40SiC composites.

This is in concordance with Figure 8a, which shows the relative piston travel vs. time curves of the studied composites. From this figure, it is possible to observe that compaction of 80SiC started later in all experiments (at 1460 s) and ended after 382 s, having a total sintering duration of 1842 s.

Yan et al. [65] reported that TiB_2_ particles are always covered by B_2_O_3_ and TiO_2_ oxide layers during their production, which evaporate (B_2_O_3_) or preserve their solid state (TiO_2_) at high temperatures. Moreover, the authors indicated that some chemical reactions (1–5 in Table 4) that can help and accelerate the sintering process can be induced due to the presence of these oxides.

In addition, Fattahi et al. [55] indicated that B_2_O_3_ can be evaporated under external pressure and at a temperature below 1600 °C (reaction 6), and B_4_C can react with TiC to generate TiB_2_ and carbon (reaction 7), which can then react with TiO_2_ to produce TiC again (reaction 8) [66].

In the present work, no traces of oxides were identified in the XRD patterns of raw and sintered materials. This may be related to their low concentrations or the formation of an amorphous structure. Assuming a low presence of these oxides, reactions 1–5 can take place, generating gaseous, such as CO and SiO, and solid compounds, such as TiC, TiB_2_, B_4_C, and SiO_2_. Moreover, it is very possible that in our study, SiO_2_ formed an amorphous structure between the grains that could not be identified by XRD analysis. Figure 8b shows the relative piston travel vs. temperature curves of the studied composites, and it demonstrates that for 80SiC, the piston remains in one position between 1050 °C and 1660 °C, and then it begins smoothly moving up, indicating the start of sintering, in which the reactions of Table 4 take place.

During the SPS of the 40SiC composite, the matrix of which consists of conductive phases TiB_2_ and TiC, DC pulses flow through the graphite punches and powder, bypassing the graphite matrix (Figure 7c). In this case, heat is generated by two different mechanisms: plasma and Joule heating [67,68,69]. The first takes place when a micro-spark discharge occurs in the gap between neighboring powder particles, generating localized and momentary heating of the particle surfaces up to several thousand degrees Celsius. Since the micro-spark discharges have a uniform distribution throughout the powder volume, the heat generated will also have a uniform distribution. Joule heating occurs when DC passes through the conducting particle and flows from the particle to its conducting neighbor through their mutual contact point.

Based on all of the above data, and taking into account the thermal properties of the predominant phases, the sintering process of 40SiC should take less time compared to the other samples. This is in agreement with the graphs in Figure 8a, in which it is seen that this material reaches a temperature of over 1650 °C degrees in 1300 s, which is 2.67 min earlier than that for the sintering of 80SiC. During compaction, the piston moves for 374 s, which is very close to the time shown for 80SiC. The relative piston travel vs. temperature curves of 40SiC and 80SiC (Figure 8b) are very similar in the interval 1650–1910 °C, showing that the reactions indicated in Table 4 also take place. Interestingly, the curves of these two materials closely coincide in the interval 1910–2000 °C, indicating that the sintering rate is the same. Moreover, [70] mentioned that the combination of TiB_2_ and TiC is thermodynamically stable up to 2427 °C, undergoing a quasibinary eutectic reaction in the temperature range 1627–1727 °C. In addition, the authors argue that for compositions other than a eutectic composition, the eutectic liquid that forms at the TiB_2_ and TiC particles is transient in nature. Since the eutectic liquid has very good wettability with both TiB_2_ and TiC, it spreads over the solid particle surfaces shortly after its formation due to surface tension. This explanation, in combination with the presence of the Joule heat, which increases the diffusion of atoms/molecules and enhances their growth, may explain the high and medium grain growth (Figure 6) for 40SiC and 80SiC, respectively. It should be noted that the presence of a large amount of eutectic liquid in the 40SiC composite material under the applied pressure will lead to the compression of the liquid phase in the voids and a reduction in the number of pores. This can explain why the density of 40SiC is higher than that of 80SiC, a compound that has a lower amount of TiB_2_-TiC phases (Figure 2).

We consider that the compaction of 60SiC composite by SPS is a combination of the two previously proposed sintering variants, since the conductive and semiconducting phases are present in almost the same proportions. In this case, we assume that heat is formed both in the dies and pistons, as well as within the material (Figure 7b), but in smaller quantities compared to the 80SiC and 40SiC processes, respectively.

From Figure 8a, it can be seen that the sintering process of 60SiC is the longest. In addition, it is observed that the piston begins its upward movement at 1328 s with a temperature of 1466 °C, reaches 2000 °C after 437 s, and ends its displacement at 1943 s. During compaction, the movement of the piston lasts for 477 s, which is 27.5% and 25% longer compared to the times observed for 40SiC and 80SiC, respectively. It seems that the combination of Joule heating in the matrix and the presence of the micro-spark discharge in the gap between particles leads to slow heating (at 1328 s, the 40SiC sample had already reached 1675 °C) that favors the formation of SiO_2_ at the grain boundaries of the particles (Table 4, reaction 1). This, in turn, forms a barrier that prevents the growth of particles. Furthermore, SiC particles mostly form a barrier between the TiC and TiB_2_ particles, which also prevents grain growth (Figure 5b). Figure 8b shows that the displacement curve of 60SiC coincides with 80SiC in the range of 1650–1900 °C, indicating that sintering occurs with the same speed. After 1900 °C, the sintering rate of 60SiC is reduced and remains constant until reaching 2000 °C.

This shows an unusual behavior, which needs further investigation to determine which sintering mechanisms occur in this material.

### 3.3. Mechanical Property Characterization

**Figure 9:**
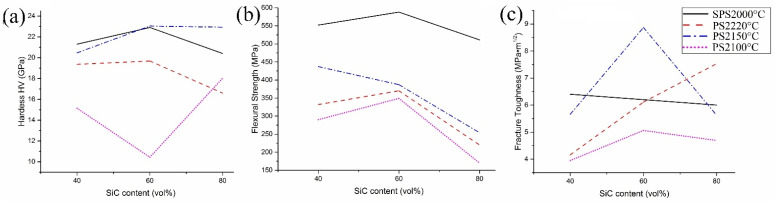
Mechanical properties of SiC-TiB_2_-TiC composites sintered by SPS and PS: (**a**) Hardness, (**b**) Flexural Strength, and (**c**) Fracture toughness vs. SiC content (vol.%).

The results obtained in this study were compared with previously published mechanical properties of SiC-TiB_2_-TiC composites sintered at different temperatures by PS (Figure 9).

Figure 9a shows the hardness of SiC-TiB_2_-TiC composites obtained by SPS and PS. Comparing the presented data, it can be seen that the hardness curves do not have an explicit behavior. The results of SPS2000 °C and PS2220 °C show a trend very similar to that obtained in this work, in which the hardness varies according to the composition. In both cases, 60SiC was the hardest, with 22.9 GPa and 19.68 GPa for SPS and PS, respectively. The hardnesses of SPSed 40SiC and 80SiC were 21.3 and 20.4 GPa, whereas 19.36 and 16.59 were obtained for composites produced by PS, respectively. These values are directly related to the grain size (Figure 6) and the density (Figure 2) of the samples, where the smaller the grain size and the greater the density, the greater the hardness observed. The PS2150C and PS2100C results have trends that differ greatly from their relative density curves (Figure 2), but the lack of a discussion regarding this result prevents an objective understanding of the reason for this behavior.

The SPSed 60SiC composite was found to have higher strength (588 MPa) than 80SiC (511 MPa) and 40SiC (552 MPa) composites. The higher value of 60SiC is explained by its fine-grained and homogeneous structure, whereas the lower values of 80SiC and 40SiC composites can be related to the higher porosity of these materials. Moreover, because of a more homogeneous structure and smaller grain size, there is a high number of strong SiC–TiC and TiB_2_–TiC interactions, which also leads to improved strength values and compensates for the possible reduction due to the no or little interaction between SiC and TiB_2_ particles [5,71,72]. On the other hand, the samples obtained by PS at 2100 °C and 2220 °C have the same tendency as the SPSed composites but with lower strength values in the range of 40–70%. In the case of PS2150 °C, the tendency is the same for 60SiC and 80SiC; however, for 40SiC, the strength is higher than expected.

Figure 9c shows the fracture toughness of the SPSed and PSed composites. Here, it can be seen that the composites have different trends. For samples obtained by SPS, the trend is a decreasing line in which the lowest, intermediate, and highest values are obtained by 80SiC (6.0 MPa∙m^1/2^), 60SiC (6.2 MPa∙m^1/2^), and 40SiC (6.4 MPa∙m^1/2^), respectively. This can be mainly explained by the amount of the tougher phase (TiB_2_) in the composite and the possible formation of amorphous SiO_2_ between the grains during compaction. In 40SiC, the largest volume of TiB_2_ is found, which can form much more amorphous SiO_2_ during sintering, according to reaction 1 in Table 4. The opposite could have occurred with 80SiC, where 80% of the volume is SiC. This is why these two composites show extreme values, but, despite the different ratios of the phases, the average values of fracture toughness are similar (around 6.2 ± 0.2 MPa·m^1/2^). Moreover, for the three compounds, the inconsistency of the CTE of TiB_2_, TiC, and SiC results in significant tensile stresses at the grain boundaries and compressive stresses in the matrix.

**Figure 10:**
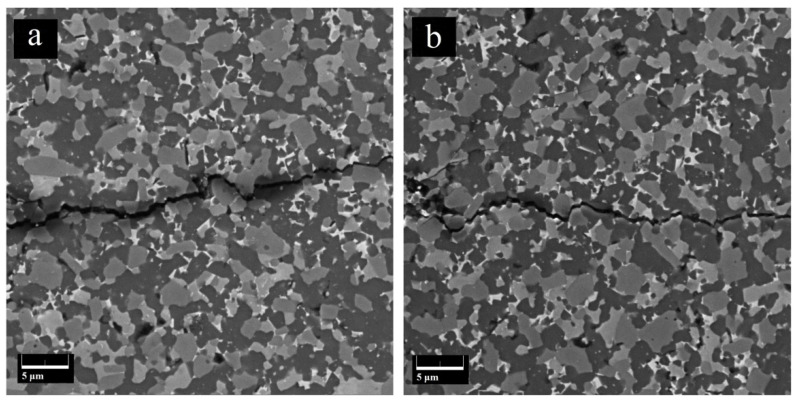
Toughening mechanisms on the crack path in sample 60SiC: (**a**) crack deflection + interface debonding; (**b**) crack deflection + crack branching.

Figure 10a,b show the crack paths in the 60SiC composite created by the Vickers indentation test. The fracture toughness of this composite is explained by various toughening mechanisms. For instance, crack deflection mainly at TiB_2_ and TiC grains, crack branching, and interface debonding were noted as mechanisms of toughening.

In these figures, a mixed transgranular and intergranular fracture mode of SiC grains can be observed. When the propagating crack encounters SiC particles, the transgranular fracture mode takes place, and then the crack can be deflected into the weak SiC-TiB_2_ grain boundaries or where there is amorphous SiO_2_. Therefore, more fracture energy can be dissipated, resulting in improved fracture toughness of the composite material.

Based on the obtained results, the composite with the best mechanical properties was identified as 60SiC.

## 4. Conclusions

The SiC-TiB_2_-TiC composites were sintered at 2000 °C in a vacuum under a pressure of 80 MPa for 3 min. The microstructures and mechanical properties of sintered samples were analyzed.

The main results are as follows:

- It was demonstrated, for the first time, that consolidation of the SiC-TiB_2_-TiC system by SPS strongly affects the properties of the composite, depending on the electrical conductivity of the raw powder mixture.
- When the matrix consists of 80 vol.% SiC (semiconductor material), sintering proceeds as conventional hot pressing, where heat is generated in the graphite matrix and then transferred to the powder. The mechanical properties of this composite were the lowest obtained in this study, and its relative density was 98.2% after sintering.
- When the matrix consists of conductive materials (37.5TiB_2_-22.5TiC, vol.%), DC pulses flow through the graphite punches and powder, bypassing the graphite matrix, and heat is generated in the powder by plasma and Joule heating. This reduces the process time compared to the other studied composites. These composites showed large grain sizes, but the density (98.9%) and mechanical properties were higher than those of the composite with a semiconductor matrix.
- The sintering of the 60SiC-25TiB_2_-15TiC composite showed that heat is generated in the graphite die and the powder in parallel. Therefore, this material underwent faster heating up to 1470 °C, and then its consolidation began. The behavior of the consolidation differed from that of the other composites. This phenomenon requires further research to clarify the reason that it occurs.
- The 60SiC composite reached the highest relative density (99.8%) and showed a fracture toughness of 6.2 MPa·m^1/2^ as well as the highest values of flexural strength (588 MPa) and hardness (23 GPa) due to more a homogeneous structure and smaller grain size.
- In the 60SiC composite, crack deflection at TiB_2_ and TiC grains, crack branching, and interface debonding were noted as the main toughening mechanisms. Additionally, all composites showed a mixed transgranular and intergranular fracture mode in SiC grains.

Based on the presented results, it can be concluded that SPS, with the parameters used in this work, is a promising sintering method for the 60SiC-25TiB_2_-15TiC vol.% composite. Further research is needed to clarify the sintering mechanism observed for the 60SiC composite.

## Figures and Tables

**Table 1:** Properties of SiC, TiC, and TiB_2_ ceramic materials at room temperature.

	SiC [29] *	TiB_2_ [30] *	TiC [31] *
**Physical properties**			
Density (g/cm^3^)	3.1	4.5	4.91
**Mechanical properties**			
Fracture toughness, K_IC_ (MPa·m^1/2^)	4.0 [30]	6.2	4.4 [5]
Vickers hardness (GPa)	27.4	25	28–35
Young’s modulus (GPa)	476 [30]	565 [32]	410–510
Shear modulus (GPa)	165 [33]	255	186
Flexural strength (MPa)	500 [5]	890 [5]	550 [5]
Poisson’s ratio	0.140	0.108	0.191
**Thermal properties**			
Melting temperature (°C)	2830	3189 [32]	3067
Thermal shock resistance ΔT (°C)	500 [30]	110 [34]	460 [35]
Thermal conductivity (W/m·K)	120	96	26 [36]
Thermal diffusivity (mm^2^/s)	~50 [33]	~29 [37]	7.5 [38]
Specific heat (J/Kg·K)	750	870 [33]	840 [36]
Thermal expansion (×10^−6^/°C)	5.12 [33]	7.4	7.6
**Electrical properties**			
Electrical conductivity (S/cm)	0.7 × 10^−4^ [39]	~10^5^ [40]	30 × 10^6^
Electrical resistivity (μΩ·cm)	10^8^–10^12^	15 [32]	68

* Generally, the data for each column were taken from the indicated sources. When the necessary information was missing, it was found in other sources, which are indicated next to the corresponding values.

**Table 2:** Raw materials information.

Material	Particle Size, d_50_ (μm)	Purity, %	Manufacturer
SiC	0.6	96–99.9	“Plasmotherm” Ltd., Moscow, Russia
TiC	0.5	>99.5	“Plasmotherm” Ltd., Moscow, Russia
TiB_2_	0.9	99.9	“Plasmotherm” Ltd., Moscow, Russia

**Table 3:** Composition of ceramic composites.

Material	Content of Each Component (vol.%)			Theoretical Density (g/cm^3^)
	SiC	TiB_2_	TiC	
80SiC	80	12.5	7.5	3.51
60SiC	60	25.0	15.0	3.77
40SiC	40	37.5	22.5	4.03

**Table 4:** Possible chemical reactions during the sintering of the SiC-TiB_2_-TiC system.

Number	Chemical Reaction	Temperature	
1	SiC + TiO_2_ = TiC + SiO_2_	from 20 °C to 2000 °C	[65]
2	B_2_O_3_ (s) = B_2_O_3_ (l)	at 450 °C	[65]
3	3SiC + 2TiO_2_ = 2TiC + CO(g) + 3SiO (g)	from 1400 to 1600 °C	[65]
4	2B_2_O_3_ (l) + 2TiO_2_ + 5SiC = 2TiB_2_ + 5CO (g) + 5SiO (g)	at 1620 °C	[65]
5	7SiC + 4B_2_O_3_ (l) = 2B_4_C + 7SiO (g) + 5CO (g)	at 1635 °C	[65]
6	B_2_O_3_ (l) = B_2_O_3_ (g)	below 1600 °C (*)	[55]
7	B_4_C + 2TiC = 2TiB_2_ + 3C (s)	from 20 °C to 2000 °C	[55]
8	TiO_2_ (s) +2C (s) = TiC (s) + 2CO (g)	from 20 °C to 1900 °C	[66]

* Under pressure.

## Data Availability

The data described in this article are openly available in previous works.
